# Comparison between Y90 Radioembolization Plus Sorafenib and Y90 Radioembolization alone in the Treatment of Hepatocellular Carcinoma: A Propensity Score Analysis

**DOI:** 10.3390/cancers12040897

**Published:** 2020-04-07

**Authors:** Antonio Facciorusso, Irene Bargellini, Marina Cela, Ivan Cincione, Rodolfo Sacco

**Affiliations:** 1 Gastroenterology Unit, University of Foggia, 71100 Foggia, Italy; estermarinacela@gmail.com (M.C.); r.sacco@ao-pisa.toscana.it (R.S.); 2 Department of Interventional Radiology, Pisa University Hospital, 56124 Pisa, Italy; irenebargellini@hotmail.com; 3 Department of Clinical and Experimental Medicine, University of Foggia, 711000 Foggia, Italy; i.cincione@unifg.it

**Keywords:** TARE, HCC, loco-regional treatment, survival

## Abstract

Background: Adjuvant sorafenib may enhance the efficacy of transarterial radioembolization with yttrium-90 in hepatocellular carcinoma patients. The aim of this study is to assess the efficacy and safety of radioembolization plus sorafenib in comparison to radioembolization alone. Methods: Out of 175 hepatocellular carcinoma (HCC) patients treated with radioembolization between 2011 and 2018, after propensity score matching, two groups were compared: a group of 45 patients that underwent radioembolization while being on sorafenib (Group 1) and a second group of 90 patients that underwent radioembolization alone (Group 2). Results: Baseline characteristics of the two groups were well balanced concerning liver function and tumor burden. No significant differences in survival outcomes were identified (median overall survival 10 vs. 10 months; *p* = 0.711), median progression-free survival 6 vs. 7 months (*p* = 0.992) in Group 1 and Group 2). The objective response rate in Group 1 vs. Group 2 was 45.5% vs. 42.8% (*p* = 1) according to mRECIST. No differences in toxicity nor in liver decompensation rates were registered. Conclusions: The association of sorafenib does not prolong survival nor delay progression in patients treated with radioembolization. Liver toxicity does not differ among the two therapeutic schemes.

## 1. Introduction

Hepatocellular carcinoma (HCC) represents the third most common etiology of cancer-related mortality and the most frequent cause of death in cirrhotic patients [1,2]. Due to the recent implementations of surveillance protocols, diagnostic algorithms and therapeutic tools, HCC is currently diagnosed in early stage in 30–60% of cases [3]. However, a considerable number of subjects still develop tumoral portal vein thrombosis (PVT) due to HCC recurrence or progression [4], thus falling in an advanced tumoral stage not suitable for radical treatments.

Patients with HCC and PVT generally have a consistent derangement of synthetic function, and a consequent precarious liver compensation that prevents any attempts of surgical cure. Furthermore, in the presence of PVT, arterial embolization procedures can increase the risk of liver failure; therefore, the presence of PVT is generally considered a contraindication to transarterial chemoembolization (TACE) [2,5,6].

Two landmark phase III trials [7,8] showed a survival benefit in advanced HCC patients treated with sorafenib, an oral multi-tyrosine kinase inhibitor, and such survival advantage has been confirmed in the analysis of the subgroup of patients with PVT [9]. Sorafenib has been therefore established as the standard of care for treatment of advanced HCC by both the American Association for the Study of Liver Diseases (AASLD) [10] and the European Association for the Study of the Liver (EASL) [2].

Yttrium-90 radioembolization (Y90RE), a well-established form of liver-directed brachytherapy, has gained an increasing role in the management of unresectable HCC in the last decade, demonstrating its efficacy by inducing necrosis and delaying tumor progression [11,12,13,14,15]. Unlike other transarterial therapies, Y90RE is a microembolic procedure that does not significantly alter the hepatic arterial flow, and its efficacy and safety have also been demonstrated in patients with HCC and PVT, with patient outcomes and tumor control rates competitive with respect to the standard of care sorafenib [16,17].

It is well known that intrahepatic locoregional therapies, such as TACE and Y90RE, may produce ischemia, thus enhancing the release of vascular endothelial growth factor (VEGF), which can trigger tumor angiogenesis [6]. Considering that the multikinase inhibitor sorafenib is thought to act directly on VEGF receptors, there could be a strong rationale in combining such therapy to Y90RE in order to counteract the VEGF effect. Moreover, it has been reported that more than 50% of patients progressing after Y90RE are not eligible for sorafenib because of poor liver function [18]: hence, it could be useful to start the therapeutic course with a combined therapy of sorafenib and loco-regional treatments (LRTs) aimed at treating patients effectively when they are amenable to treatment (i.e., with good liver function) rather than at tumor progression.

The recent multicenter SORAMIC trial failed to find a significant superiority of the combination Y90RE plus sorafenib versus sorafenib alone although subgroup analyses indicated a survival benefit of combo therapy in patients without cirrhosis, cirrhosis of non-alcoholic aetiology, or patients ≤65 years old [19]; moreover, a higher rate of adverse events was observed with the combined approach [20]. On the other hand, comparative studies between Y90RE plus sorafenib and Y90RE alone are lacking.

The aim of this study is to evaluate the efficacy and the safety profile of the combined therapy of sorafenib with Y90RE in comparison to Y90RE alone for the management of unresectable HCC.

## 2. Results

### 2.1. Study Groups and Balancing

**Figure 1:**
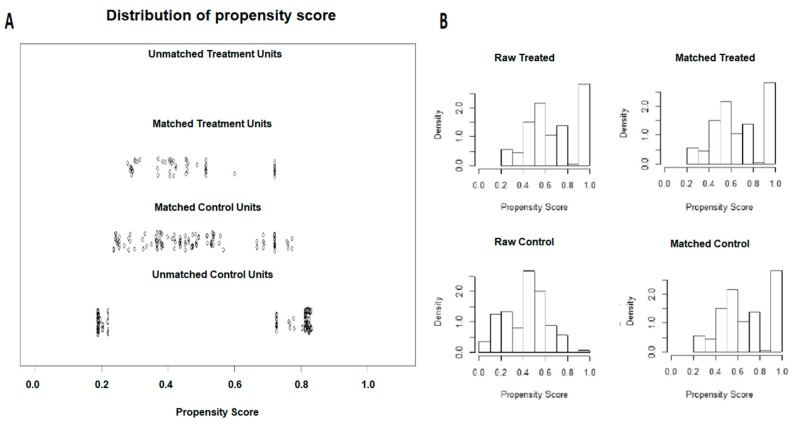
Propensity score matching of the two study groups. Out of the initial 175 patients, after 1-to-2 propensity score caliper matching 135 patients were included in the study: 45 treated with radioembolization plus sorafenib and 90 treated with radioembolization alone. (**A**) Propensity score matching jitter plot; (**B**) propensity score matching histogram.

Out of 175 HCC patients initially identified (60 in Group 1 and 115 in Group 2), after 1-to-2 propensity score match, 135 patients were selected for comparison: 45 HCC patients who underwent Y90RE plus sorafenib and 90 subjects treated with Y90RE alone (Figure 1). The characteristics of the 135 propensity score-matched patients are reported in Table 1.

The median age in both groups was 62 years, with a prevalence of men (73.3% and 80% in Group 1 and 2, respectively) and with hepatitis C virus (HCV) as the predominant etiology of the underlying liver disease (46.7% and 42.2%, respectively). Most patients were in Child Pugh (CP) A stage (only two CP B patients in Group 2). Tumor burden was more than 50% of liver volume in 13.3% vs. 15.4% of patients in Group 1 and Group 2 respectively, whereas median alpha-fetoprotein (AFP) was 40.8 ng/mL in Group 1 and 48.2 ng/mL in Group 2. The Barcelona Clinic Liver Cancer (BCLC) stage was B in 20% and C in 80% of patients in both groups, reflecting the absence/presence of portal vein thrombosis (PVT). The groups appeared to be statistically balanced for all the characteristics taken into consideration.

Patients had undergone previous locoregional or surgical treatments before 90YRE in 73.3% vs. 80% of patients in Group 1 and Group 2, respectively (*p* = 0.37). In particular, in Group 1, 25 patients had undergone TACE (20 patients two TACE sessions and 5 patients 3 TACE sessions) and eight patients radiofrequency ablation (RFA); on the other hand, out of 72 patients with previous treatments in Group 2, 58 underwent TACE (two TACE sessions in 50 patients and three TACE sessions in eight subjects) and 14 patients underwent RFA. In Group 1, the median time on sorafenib before 90YRE was 2 months (range 1–6), and no patient had a radiological demonstration of progression nor a toxicity that contraindicated prosecution of such therapy. For this reason, patients in Group 1 continued with sorafenib therapy also after 90YRE until any reason for treatment withdrawal (such as tumor progression or liver decompensation) occurred. The median overall time on sorafenib therapy for patients in Group 1 was 9 months, with a median dosage of 800 mg daily (range 200–800). Y90RE was performed in a single session in 18 patients in Group 1 and 49 patients in Group 2; the other patients underwent two sessions. None of the recruited patients underwent more than two Y90RE sessions.

### 2.2. Survival Analysis

The median follow-up for Group 1 and Group 2 was 7 (95% CI: 6–20) and 11 (95% CI: 8–19) months, and 18 and 42 deaths were registered in each group, respectively. The causes of death in Group 1 and Group 2 were tumor progression in six (33.3%) and nine (21.4%) cases (*p* = 0.737), liver failure in nine (50%) and 27 (64.3%) cases (*p* = 0.475) and non-liver/non-tumor events in three (16.7%) and six (14.3%) cases (*p* = 0.89); thirty-days mortality was 16.7% and 7.1%, respectively, and in all cases appeared to be tumor related.

**Figure 2:**
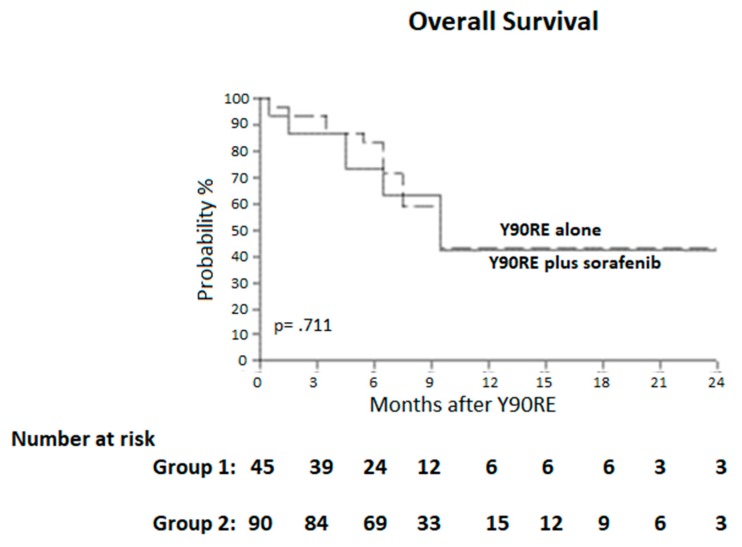
Overall survival curves. Median survival was 10 months (7–12) and 10 months (8–11), in the two groups. Y90RE, Yttrium 90 radioembolization.

Two-years overall survival and median survival (95% CI) of patients in Group 1 and Group 2 were 41.9% and 10 months (7–12) vs. 42.6% and 10 months (8–11), with no differences between groups (*p* = 0.711) (Figure 2). Subgroup analysis restricted to BCLC C patients (i.e., patients with PVT) confirmed the above-reported results; in particular, the median survival was 8 months in Group 1 and 7 months in Group 2, with no difference between the two groups in the subset of subjects with PVT (*p* = 0.76). On the other hand, BCLC B patients showed a median survival of 12 months in both groups (*p* = 0.88).

**Figure 3:**
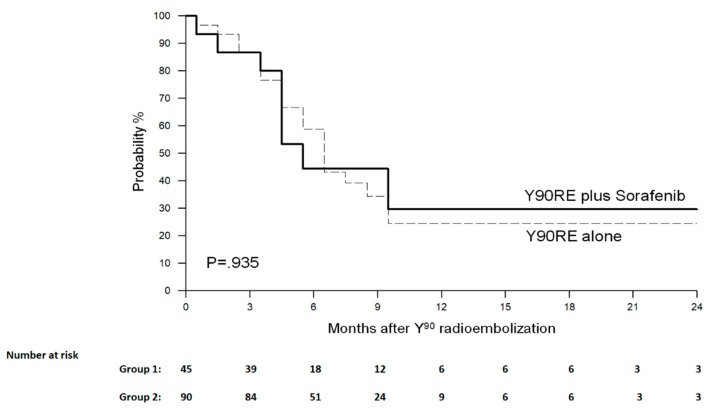
Progression-free survival curves. Median progression-free survival was 6 months and 7 months in the two groups, respectively (*p* = 0.992).

During the study follow-up, 15 progressions (of which nine cases of extrahepatic metastases) were observed in Group 1 and 33 (of which 21 cases of extrahepatic spread) in Group 2. The progression-free survival (PFS) at 2 years and the median PFS in Group 1 vs. Group 2 were 29.6% and 6 months vs. 24.7% and 7 months (*p* = 0.992) (Figure 3). At tumor progression, Child Pugh status in Group 1 vs. Group 2 was A in 12 patients (80%) vs. 25 (76%) and ≥ B7 in three patients (20%) vs. eight (24%) (*p* = 0.397). At progression, three (20%) and nine (27.2%) patients started regorafenib in the two groups, respectively, whereas the other patients underwent only the best supportive care.

### 2.3. Tumor Response Evaluation

**Figure 4:**
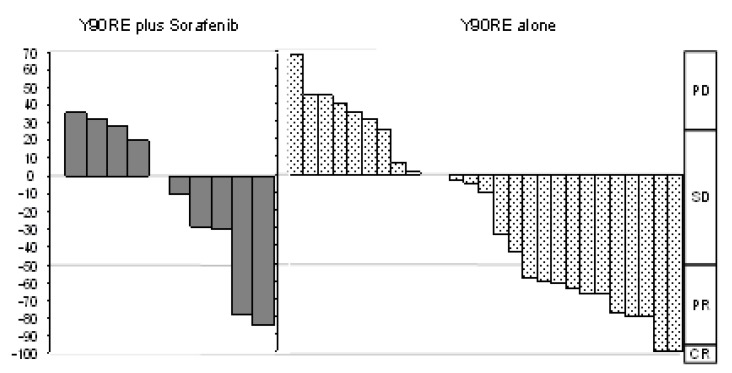
Waterfall graphic representing the tumor response rate in the two groups. CR, complete response; PD, progressive disease; PR, partial response; SD, stable disease.

The objective response (OR) in Group 1 vs. Group 2 was 27.3% vs. 35.7% (*p* = 0.71) according to RECIST 1.1 and 45.5% vs. 42.8% (*p* = 0.89) according to mRECIST. The best response described through mRECIST criteria is depicted in Figure 4. The disease control rate (DCR) to Y90RE in Group 1 vs. Group 2 was 54.54% vs. 71.4% (*p* = 0.45) according to RECIST 1.1 and 54.54% vs. 75% (*p* = 0.26) according to mRECIST.

### 2.4. Y90RE Toxicity and Liver Decompensation

Details of treatment-related toxicities are reported in Table 2. Among post-Y90RE clinically relevant toxicities recorded within 3 months from treatment, the most common included ascites (12 (8.9%)), abdominal pain and nausea (6 (4.4%)); no difference in the incidence of such events was recorded between the two groups. At laboratory sampling, bilirubin was the most common altered sampling affecting 11.1% of the patients, followed by albumin in 6.6% and lymphocyte count in 6.6%. Again, no significant differences in laboratory toxicity were registered within the two study groups. Within 3 and 6 months from therapy, 46.7% and 33.3% of the patients in the two groups experienced at least one episode of liver decompensation, with no difference among the two groups of patients. All patients with liver decompensation were hospitalized and managed according to the standard of care (diuretics and albumin in the case of ascites, non-absorbable disaccharides for encephalopathy).

## 3. Discussion

Treatment options in patients with unresectable HCC are very limited. Although both sorafenib and Y90RE have shown efficacy in advanced HCC, tumor progression is common and long-term survival rates are low [6,7,8,10].

The combination treatment with sorafenib and LRTs has a strong scientific rationale, extensively clarified in the case of transarterial chemioembolization [21]. In fact, LRTs induce tumor ischemia, resulting in a slight increase in VEGF and other hypoxia-induced factors, whereas the well-known antiangiogenetic activity of sorafenib inhibits specific receptors of such molecules [22,23]. Moreover, in the case of Y90RE, the antiangiogenetic effect of sorafenib could theorically enhance its efficacy by reducing eventual atero-venous shunts, as suggested by some preliminary reports [24].

Despite the disappointing results of a large preliminary randomized controlled trial (RCT) [19], further studies are needed to properly evaluate the possible synergistic effects of the association of sorafenib and Y90RE. In particular, evidence on the comparison between Y90RE plus sorafenib versus Y90 alone is scarce, mainly provided only by a single RCT conducted in the pre-transplant setting, hence without the assessment of long-term survival outcomes [25,26].

In attendance of the results from the ongoing STOP-HCC trial, conducted by the same American group [27], our study represents the first series comparing the two treatment approaches and reporting long-term survival and progression outcomes.

From a prospectively collected database, we matched through propensity score analysis 45 patients (Group1–combination treatment) that had been previously started sorafenib and then were treated with Y90RE with a second group of patients (Group 2–control group) that had been treated only with Y90RE. The two groups appeared to be very well balanced in terms of demographics, liver function and tumor burden (Table 1), making the comparison between the two treatment strategies meaningful.

We did not register a significant difference in survival outcomes between the two groups, with the median OS (95% CI) being 10 months (7–12) vs. 10 months (8–11) in Group 1 and Group 2 respectively (*p* = 0.711). Similarly, the association of sorafenib and Y90RE did not result in a clear advantage in terms of progression-free survival being median PFS 6 vs. 7 months in Group 1 and 2 respectively (*p* = 0.992). These results confirm the findings of the aforementioned SORAMIC trial [19], thus strengthening the concept of the lack of benefit from combining the two treatments.

Since LRTs increase the levels of hypoxia-induced factors, such as VEGF, and due to the well-known antiangiogenetic and cytostatic activity of sorafenib, the combination therapy has the theoretical potential to synergically improve the efficacy in decreasing tumor burden. Nevertheless, in our series, tumor response did not differ between the two groups, either when evaluated according to purely dimensional criteria (RECIST 1.1, *p* = 0.71) or when evaluated according to diameters of the viable tumor (mRECIST criteria, *p* = 1). Similarly to our results, the tumor response analysis from the aforementioned trial comparing the two treatments in the pre-transplant setting did not find any increased efficacy deriving from the combination treatment [28].

It has been reported that more than 50% of patients progressing after Y90RE are not eligible to sorafenib because of poor liver function [18]: this finding stands for the proposal of a combined therapy in CP A patients in order to effectively treat them with all the possible armamentaria when they are amenable to be treated. Our study confirmed that the majority of patients have a worsening CP class at progression after Y90RE, hence missing the chance to be offered any further therapy such as systemic drugs. This finding suggests that there is a short “therapeutic window” before progression when sorafenib or second-line therapies such as regorafenib [29] could be considered, and may support the early use of systemic agents (i.e., combined to LRTs or just before progression) at the time when liver function is maintained.

Due to the high incidence of side effects reported after both therapies, concerns may arise about the safety profile of the combined treatment. In our study, no differences were found in treatment toxicity and similar rates of liver decompensation occurred in the two groups (46.7% in Group 1 vs. 33.3% in Group 2, *p* = 0.517). This confirms the data reported in the above-cited randomized study [25] and, although further studies with broader sample size are required, it suggests that sorafenib combined with Y90RE is feasible and tolerable. Of note, 30-day mortality was significantly higher in the combined group and this may be explained with the increased early toxicity due to the synergic effect of sorafenib and Y90RE. However, as time passes after the treatment, the rate of liver decompensation tended to be homogenous between the two study groups.

Among the weaknesses to our study, there are the small sample size and the absence of randomization due to the retrospective nature of our analysis. However, a robust propensity score matching model was built aiming to obviate the potential selection biases. Second, progression-free survival could be an unreliable endpoint in HCC patients because death resulting from cirrhosis might confound detection of potential benefits from the treatment. However, since in our series most of the patients presented a well-preserved liver function, the impact of death unrelated to tumor progression has been minimized. Moreover, dosimetry was not routinely performed and this could result in possible differences between groups with regard to absorbed dose in the normal liver and tumor as well as PVT targeting. Finally, as most patients had undergone previous treatments, there is a risk of immortal bias since the follow-up was considered from the time of Y90RE. However, the proportion of subjects who underwent previous treatments was similar between the two groups, hence this aspect was unlikely to influence our findings.

With such boundaries, our study represents the first report comparing Y90RE plus sorafenib to Y90RE alone in the field of unresectable HCC and could open the way to the research of novel therapeutic schemes for advanced patients. Results of further large RCTs are warranted in order to put the findings of the current manuscript into context. As long as the results of such trials are not available, it cannot be definitively established whether combined therapy has a competitive efficacy role in these patients.

## 4. Materials and Methods

### 4.1. Patients

From a prospectively collected database, we retrieved data of 175 consecutive intermediate-advanced HCC patients undergoing Y90RE at two Italian centers between Apr 2011 and Dec 2018. We collected their demographical data, etiology of hepatopathy, previous tumoral treatments, liver function according to Child-Pugh (CP) score, tumor burden and stage (according to BCLC, Cancer of the Liver Italian Program (CLIP) and Okuda systems), portal thrombosis presence and extension. Institutional Review Board approval for this retrospective report was obtained (Committee of the Ospedali Riuniti di Foggia, OR-2314-2020).

The study population included a group of 60 patients treated with Y90RE while being on sorafenib because of previous indication from other institutions and that continued sorafenib after Y90RE (Group 1—study group), and a second group of 115 patients that underwent Y90RE alone (Group 2—controls). At our centers, Y90RE is commonly offered to patients with liver cirrhosis and HCC confined to the liver (absence of extrahepatic spread) and not eligible for conventional curative treatments (i.e., liver resection, ablative therapies or transplantation). The decision to treat patients with Y90RE is made by consensus at our weekly multidisciplinary HCC conference composed of staff from hepatology, medical oncology, surgery, and interventional radiology. There was no difference in baseline factors between the two groups.

### 4.2. HCC Diagnosis and Staging

Diagnosis of HCC was radiological or histological as per current guidelines [2,10]. Tumor-related PVT was assessed at baseline computed tomography (CT) or magnetic resonance imaging (MRI) in the case of a filling defect in the portal venous phase, with enhancement during the arterial phase of dynamic imaging, associated to HCC. PVT extension was classified as PV1 (segmentary), PV2 (left or right portal branch), PV3 (main portal trunk and PV4 (superior mesenteric vein), as previously described [30]. Tumor burden was computed as the ratio of tumor on whole liver volume. Portal hypertension was defined in the presence of varices and/or thrombocytopenia (platelet count <100,000/μL) associated to splenomegaly.

### 4.3. Y90 Procedure

Y90RE was performed in two sessions: a simulation session consisting of the injection of ^99^Tc-macroaggregated albumin into the hepatic arterial bed simulating ^90^Y microspheres distribution, in order to evaluate through SPECT scintigrams the extrahepatic deposition and tumor uptake; coiling of extrahepatic arteries was performed when needed. Two to three weeks later, the treatment was performed by the injection of resin microspheres (Sir-Spheres^®^; Sirtex Medical Europe GmbH, Bonn, Germany) labeled with ^90^Y. The median treatment activity was 1.25 GBq (range 0.35–4 GBq), with no difference between the two groups. The median dose was 100 Gy (range 90–120 Gy) [31]. All the procedures were successful, delivering the whole pre-planned dose. Patients were observed for 2 days and then discharged.

### 4.4. Sorafenib Treatment

Sorafenib dosage was started at the standard dose of 400 mg twice daily, and titrated or withdrawn in the case of toxicity graded according to common terminology criteria for adverse events (CTCAE) 5.0 [32], or tumor progression according to Response Evaluation Criteria In Solid Tumors (RECIST) 1.1 criteria [33].

### 4.5. Patient Monitoring and Imaging Response Assessment

Clinical visits were performed on an outpatient basis at months 1, 3, 6 and then every four months after Y90RE treatment. Liver decompensation (LD) was defined as the presence of any of the following: ascites, esophageal variceal hemorrhage, hepatic encephalopathy, total bilirubin >3 mg/dL, and prothrombin time international normalized ratio >2.2 [12]. Thoraco-abdominal CT scans and response assessment were performed according to the same timeframe of clinical visits.

Tumor response was assessed according to Response Evaluation Criteria in Solid Tumors (RECIST) criteria 1.1 [24] and modified RECIST (mRECIST) criteria [34]. Objective response (OR) was defined as partial response plus complete response, while the disease control rate (DCR) was defined as stable disease plus partial response plus complete response. All CT scans were independently assessed by an experienced radiologist (IB); when response evaluation was controversial, the final diagnosis was reached with a second opinion.

### 4.6. Statistical Analysis

Dichotomous features were expressed as an absolute number and percentage, and differences between groups were compared using the McNemar test. Continuous variables were expressed in terms of mean values and standard deviations if normally distributed, median and interquartile ranges if non-normally distributed and differences were tested through the Wilkoxon-rank test. All analyses were 2-tailed and the threshold of significance was assessed at ≤0.05.

To overcome biases due to the different distributions of covariates, an 1-to-2 match was created using propensity score analysis built upon a multivariate logistic regression model. The propensity score represents the probability of each individual subject to be allocated to a particular treatment given a set of known covariates [35]. The covariates considered were: age (>65 years vs. ≤65 years), sex, previous treatments, portal hypertension, CP stage (A vs. B), alpha-fetoprotein (AFP) level, etiology of liver disease (HCV versus other), tumor burden (below or beyond 50%), lobar distribution (unilobar vs. bilobar), and BCLC stage (B vs. C), PVT (present versus absent). A further covariate considered was the presence versus absence of alcoholic cirrhosis, as in the latter subgroup the combined approach gave significant results in the SORAMIC trial [19]. The predictive values were then used to obtain a 1-to-2 match by using the nearest neighbor matching within a specified caliper distance, defined as 0.2 of the standard deviation of the logit of the propensity score. [36,37]. Therefore, patients whose propensity score could not be matched due to a greater caliper distance were excluded.

Overall survival (OS) and progression-free survival (PFS) were computed from the time of Y90RE treatment and determined using the Kaplan–Meier methodology. The statistical analysis was performed using the MatchIt package in R Statistical Software 3.0.2 (Foundation for Statistical Computing, Vienna, Austria).

## 5. Conclusions

The state of the science of LRTs combined with systemic drugs is an evolving field. Our study shows that the association of sorafenib to Y90RE does not seem to modify outcomes nor safety profiles in advanced HCC patients. Further data of large randomized controlled studies are needed in order to confirm our findings and guide clinicians in therapeutical algorithms of unresectable HCC.

## Figures and Tables

**Table 1:** Baseline characteristics of enrolled patients.

	GROUP 1 Y90RE Plus Sorafenib (45 pts)	GROUP 2 Y90RE alone (90 pts)	*p* value
Age (years)			0.243
median (min, max)	62 (24, 74)	62 (32, 84)	
Gender			0.709
Male	33 (73.3%)	72 (80%)	
Female	12 (26.7%)	18 (20%)	
Previous treatments			0.37
No	12 (26.7%)	18 (20%)	
Yes	33 (73.3%)	72 (80%)	
Child-Pugh			1.0
A	45 (100%)	88 (97.7%)	
B	-	2 (2.3%)	
Portal Hypertension *			0.45
Absent	21 (46.7%)	36 (40%)	
Present	24 (53.3%)	54 (60%)	
AFP (ng/mL)			0.589
median (min, max)	40.8 (5, 169,190)	48.2 (5, 108,800)	
Etiology of liver disease			0.14
HCV	21 (46.7%)	38 (42.2%)	
HBV	12 (26.7%)	22 (24.8%)	
Other	12 (26.6%)	30 (33%)	
ECOG performance status			
PS 0	44 (97.8%)	90 (100%)	0.333
PS 1	1 (2.2%)	-	
Tumor burden			0.939
0–25%	18 (40.0%)	38 (42.2%)	
26–50%	21 (46.7%)	38 (42.2%)	
51–75%	6 (13.3%)	14 (15.4%)	
Lobar distribution			0.709
Unilobar	33 (73.3%)	72 (80%)	
Bilobar	12 (26.7%)	18 (20%)	
			
BCLC			1.0
B	9 (20%)	18 (20%)	
C	36 (80%)	72 (80%)	
Clip score			0.405
<2	21 (46.7%)	38 (42.2%)	
≥3	24 (52.3%)	52 (57.8%)	
PVT			0.983
absent	9 (20.0%)	18 (20.0%)	
I–II	18 (40.0%)	36 (40.0%)	
III (a/b)	15 (33.3%)	30 (33.3%)	
IV	3 (6.7%)	6 (6.6%)	
PVT			1.0
absent	9 (20%)	18 (20%)	
present	36 (80%)	72 (80%)	

Values are expressed as number (percentage) or median (ranges) where specified. *Portal hypertension is defined by the presence of a platelet count below 100,000/mm^3^ associated with significant splenomegaly, or presence of varices at endoscopy. Abbreviations: AFP, α-fetoprotein; ECOG, Eastern Cooperative Oncology Group; ALTSG, American Liver Tumor Study Group; BCLC, Barcelona Clinic Liver Cancer; CLIP, Cancer of the Liver Italian Program; HBV, Hepatitis B Virus; HCV, Hepatitis C Virus; PS, Performance Status; PVT, Portal Vein Thrombosis; Y90RE, Yttrium 90 Radioembolization.

**Table 2:** Cumulative toxicity analyses. Only grade 3–4 clinical and laboratory toxicities (CTCAE v. 5.0) recorded at 3 months are reported.

Characteristics	OVERALL	GROUP 1 Y90RE Plus Sorafenib (45 pts)	GROUP 2 Y90RE Alone (90 pts)	*p*
Clinical toxicities				
Fatigue	3 (2.2%)	3 (6.7%)	-	0.333
Abdominal pain	6 (4.4%)	3 (6.7%)	3 (3.3%)	1.000
Nausea/vomiting/anorexia	6 (4.4%)	3 (6.7%)	3 (3.3%)	1.000
Fever	3 (2.2%)	-	3 (3.3%)	1.000
Ascites	12 (8.9%)	6 (13.3%)	6 (6.7%)	0.591
Variceal haemorrhage	-	-	-	
Cholecystitis	-	-	-	
Laboratory toxicities				
Bilirubin	15 (11.1%)	3 (6.7%)	12 (13.3%)	0.651
Albumine	9 (6.6%)	6 (13.3%)	3 (3.3%)	0.254
Lymphocyte count	9 (6.6%)	3 (6.7%)	6 (6.7%)	1.000
	
Liver decompensation *	51 (37.8%)	21 (46.7%)	30 (33.3%)	0.517
				

Data are expressed in absolute number and %. * Liver decompensation is defined as the occurrence of any of the following: clinically relevant ascites, total bilirubin >3 mg/dL, hepatic encephalopathy, INR >2,2 (o Quick <40%), variceal haemorrage. CTCAE, Commont Terminology Criteria of Adverse Events
